# Immunosenescence and Inflammaging in the Pathogenesis of Non-Melanoma Skin Cancer

**DOI:** 10.3390/cancers18213481

**Published:** 2026-10-28

**Authors:** Martina Mihalj, Nika Filipović Mioč, Iva Sabljić, Zdenka Šitum Čeprnja, Stana Tokić, Marija Buljan

**Affiliations:** 1 Department of Dermatology and Venereology, Osijek University Hospital, J. Huttlera 4, HR-31000 Osijek, Croatia; mmihalj@mefos.hr; 2 Department of Dermatovenereology, Faculty of Medicine, Josip Juraj Strossmayer University of Osijek, J. Huttlera 4, HR-31000 Osijek, Croatia; 3 Department of Dermatology and Venereology, Sestre Milosrdnice University Hospital Centre, Vinogradska 29, HR-10000 Zagreb, Croatia; nika.filipovic@kbcsm.hr; 4 Department of Dermatology and Venereology, Vinkovci County General Hospital, Zvonarska ul. 57, HR-32100 Vinkovci, Croatia; iva.sabljic@obvk.hr; 5 Department of Dermatology and Venereology, University Hospital Split, Šoltanska 2, HR-21000 Split, Croatia; zsitum@kbsplit.hr; 6 Department of Laboratory Medicine and Pharmacy, Faculty of Medicine, Josip Juraj Strossmayer University of Osijek, J. Huttlera 4, HR-31000 Osijek, Croatia; stokic@mefos.hr; 7 School of Dental Medicine, University of Zagreb, Gundulićeva 5, HR-10000 Zagreb, Croatia

**Keywords:** non-melanoma skin cancer, basal cell carcinoma, cutaneous squamous cell carcinoma, immunosenescence, inflammaging, cellular senescence, skin microbiome, tumour immunosurveillance, skin aging, chemoprevention

## Abstract

Non-melanoma skin cancer (NMSC) is the most frequently diagnosed malignancy worldwide, and its incidence rises disproportionately with age. While cumulative ultraviolet (UV) exposure is the dominant recognised risk factor, it does not fully account for the steep age-associated increase in NMSC, suggesting that additional, underrecognised mechanisms operate in older skin. This review synthesises evidence that immune senescence (the age-related remodelling of innate and adaptive immunity) is a central and partly modifiable driver of NMSC aetiopathogenesis. We first outline the classical risk factors and the epidemiological and economic burden of NMSC, and revisit the proposal to regard recurrent NMSC as a chronic disease. We then examine four interlocking, age-associated processes: the accumulation of senescent cells and their pro-inflammatory secretory phenotype (“inflammaging”) in the skin microenvironment; UV-induced suppression of cutaneous immune function; age-related dysbiosis of the skin microbiome and its impact on antitumour immunity; and the systemic decline of T-cell-mediated tumour immunosurveillance. We integrate these processes into a framework in which immune senescence contributes to the progression of accumulated cutaneous damage to overt malignancy, and consider the implications for prevention (including senotherapeutics, microbiome modulation, and immune-stratified surveillance) that extend beyond UV-directed measures alone.

## Simple Summary

Non-melanoma skin cancers (basal cell and squamous cell carcinoma) are the most common cancers in humans, and their numbers are rising fastest among older adults. This increase is usually attributed to a lifetime of sun exposure, yet sun damage alone does not fully explain why these cancers cluster so strongly in the elderly. In this review, we examine an additional and often overlooked factor: the gradual weakening of the immune system that accompanies aging, known as immune senescence. We describe how aging skin accumulates worn-out (“senescent”) cells that fuel chronic inflammation, how ultraviolet light suppresses the skin’s immune defences, and how the community of microbes living on the skin shifts with age in ways that further weaken protection against tumours. We argue that the decline of the immune system ties these processes together and helps explain why skin cancer becomes so common in later life. Recognising immune aging as a central driver points toward new prevention strategies (beyond sun protection alone) aimed at supporting the aging immune system, which could help reduce the growing burden of these cancers in an aging population.

## 1. Introduction

**Figure 1:**
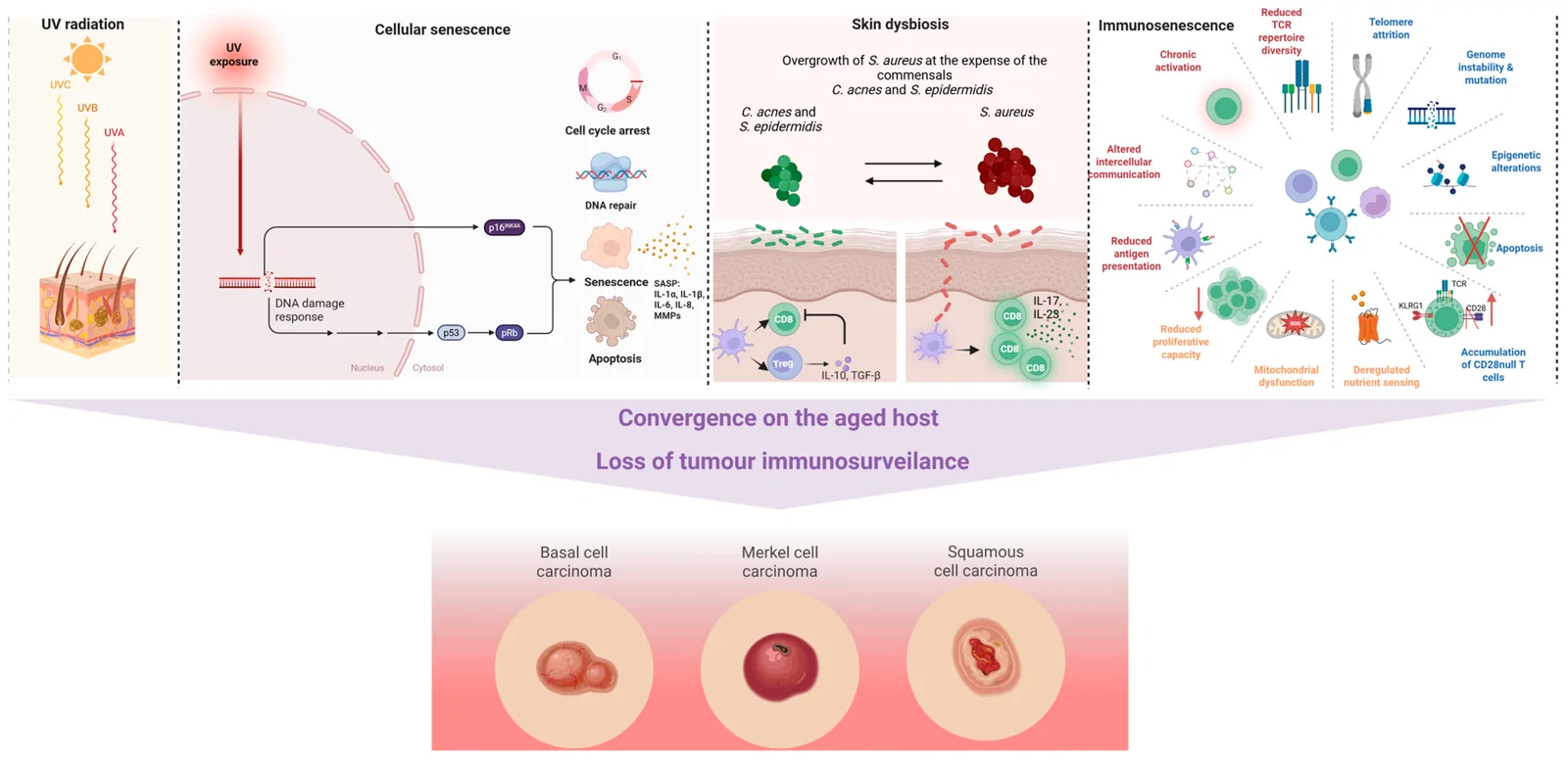
Immune senescence as the convergent driver of non-melanoma skin cancer (candidate graphical abstract). Four age-associated processes—UV radiation (direct DNA photodamage and photoimmunosuppression), cellular senescence with its pro-inflammatory secretory phenotype, skin dysbiosis, and systemic immunosenescence—converge in the aged host to produce a loss of tumour immunosurveillance and, ultimately, non-melanoma skin cancer: basal cell carcinoma, Merkel cell carcinoma, and cutaneous squamous cell carcinoma. Abbreviations: UV, ultraviolet; DNA, deoxyribonucleic acid.

Non-melanoma skin cancers (NMSCs) (comprising basal cell carcinoma (BCC), cutaneous squamous cell carcinoma (cSCC), and rarer entities such as Merkel cell carcinoma) represent the most commonly diagnosed group of malignancies worldwide. According to GLOBOCAN 2024, an estimated 1,1 million new NMSC cases and 58,721 associated deaths occurred worldwide in 2024. Within this dataset, NMSC ranked fifth by incidence, accounting for 5.5% of all newly diagnosed cancers. Because basal cell carcinoma is excluded from the incidence estimates, these figures provide only a partial representation of the overall NMSC burden [1]. The Global Burden of Disease (GBD) framework provides broader coverage by including both BCC and cSCC. The GBD 2023 analysis reported approximately 25.7 million cancers when NMSC was included and 18.5 million when it was excluded, implying approximately 7.2 million new NMSC cases worldwide in 2023 [2]. The economic burden is also substantial, with average annual expenditure on NMSC treatment in the United States estimated at US$6.5 billion during 2016–2018 [3]. More recent Australian data indicate that NMSC accounted for approximately A$1.9 billion in health system expenditure in 2023–2024, making it the third most costly cancer category nationally [4]. Since classical risk factors (cumulative ultraviolet exposure, immunosuppression, viral infection, and genetic predisposition) do not fully account for why NMSC incidence is rising fastest precisely within this aging cohort, growing attention has turned toward the age-related decline of the immune system itself, immunosenescence, as an additional and still underrecognized driver of NMSC development. This review examines the current evidence linking immunosenescence and inflammaging to NMSC pathogenesis and considers its implications for prevention in an aging population (Figure 1).

## 2. Clinical Spectrum and Established Risk Factors of Non-Melanoma Skin Cancer

Non-melanoma skin cancers encompass BCC (approximately 75–80% of cases) and cSCC (around 25%), together with rarer entities such as Merkel cell carcinoma [5]. BCC is the most common skin malignancy overall; five clinicopathologic subtypes are recognized (nodular, superficial, morpheaform, fibroepithelial, and basosquamous) which differ in anatomical distribution and clinical behaviour [6], with basosquamous carcinoma showing SCC-like aggressiveness and metastatic potential [7]. cSCC, the second most common skin cancer, typically arises from precursor lesions such as actinic keratosis or Bowen’s disease and carries a higher metastatic potential than BCC (2–6%), particularly in immunosuppressed patients [8,9]. Related entities include keratoacanthoma, regarded by some as a well-differentiated, often self-involuting variant of SCC, and verrucous carcinoma, a rare, low-grade SCC variant [10,11].

Several well-established risk factors underlie NMSC development. Cumulative and intermittent ultraviolet (UV) radiation exposure remains the principal driver, causing direct DNA photodamage and reactive-oxygen-species-mediated mutagenesis [12,13]. Ionizing radiation confers an approximately threefold increased risk of BCC and cSCC after a latency period of two decades or more [14,15]. Immunosuppression, most notably in solid-organ transplant recipients, dramatically increases NMSC incidence, up to 10-fold for BCC and 250-fold for cSCC [16,17]. Cutaneous infection with beta-genus human papillomavirus types and Merkel cell polyomavirus contributes to cSCC and Merkel cell carcinoma pathogenesis, respectively [18,19,20]. Chronic skin damage and chronic inflammatory dermatoses [21], together with air pollution [22], further add to cumulative risk. Genetic susceptibility is conferred by MC1R variants [23], hereditary syndromes such as Gorlin-Goltz syndrome [24], and rarer cancer-predisposition genodermatoses [25].

## 3. Non-Melanoma Skin Cancer as a Chronic Disease

The term “chronic disease” encompasses conditions of long duration and slow progression; the World Health Organization designates cardiovascular disease, cancer, chronic respiratory disease, and diabetes as its four principal categories [26]. Paradoxically, NMSC remains the only cancer type not currently classified as a chronic disease, despite its substantial and growing impact on patients’ quality of life, an impact that arises from the tumour itself, its treatment, and long-term sequelae [27]. NMSC is also characteristically underreported to cancer registries, which obscures its true burden and reinforces the misconception that it is a diagnosis of limited significance.

A reclassification of NMSC as a chronic disease has been proposed for high-risk individuals with multiple lesions [28]. Wehner et al. found that after an initial NMSC diagnosis, the ten-year risk of a subsequent skin tumour was 59.6% [29], indicating that a subset of patients follows a recurrent, long-term disease course consistent with the WHO’s chronic disease criteria [26]. Recognizing this subset as having a chronic disease would support the development of long-term surveillance strategies, risk-stratified follow-up, and coordinated long-term care.

## 4. Evidence for the Link Between Chronic (Skin) Diseases and NMSC Through Inflammaging

Epidemiologic data suggest a strong link between NMSCs and the immune system. As mentioned earlier, after solid-organ transplantation, the risk of NMSCs increases, with standardized incidence ratios of SCC ranging from 15 to 250 compared with the general population and BCC ratios ranging from 5–10 [16,30,31,32]. Adaptive immunity plays a vital role in the development of NMSC in this subset of patients, with CD8+ T cells being essential for effective control of tumour growth [33]. It has been postulated that the heterogeneous expression of class I HLA proteins in SCC may also explain why immunosuppression increases the SCC risk up to 250-fold, but BCC risk only 10-fold [34]. Indeed, immunosurveillance may better control SCC pathogenesis because of the partial expression of class I HLA proteins in SCC, compared with BCC, where class I HLA proteins are often completely absent [35]. Mechanistically, loss of surface HLA class I expression, through transcriptional down-regulation of the antigen-processing machinery or irreversible structural defects such as loss of heterozygosity at the HLA locus or mutation of beta-2-microglobulin (the invariant light chain required for assembly of all HLA class I allotypes), abrogates the presentation of tumour neoantigens to cytotoxic CD8+ T cells and constitutes a principal route of immune escape in squamous carcinogenesis [36]. In cSCC arising in solid-organ transplant recipients, reduced HLA class I expression has been linked to an increased risk of metastasis, and the tumour immune microenvironment is remodelled toward a permissive, growth-supporting state, indicating that under systemic immunosuppression the balance shifts further toward antigen-loss immune evasion [37,38]. Consequently, a loss of immunosurveillance due to immunosuppression would influence SCC pathogenesis more than that of BCC. Moreover, NMSCs in organ transplant patients sometimes regress after improvement in immune function, further underscoring the importance of immune surveillance in their pathogenesis [39].

The correlation of skin cancers with HIV infection, one of the most common acquired immunodeficiencies today, is less well defined. The immunosuppression of HIV undoubtedly predisposes patients to cancer development, with Kaposi’s sarcoma, some lymphomas, and cervical carcinoma classified as AIDS-defining diseases, but a recent meta-analysis reported that the HIV-infected population also has a 2-fold increased risk of developing BCC and a 5-fold increased risk for SCC compared to the general population [40]. In addition, the use of combined antiretroviral therapy may protect HIV patients against NMSC development [41]. The risk of NMSC, especially SCC, increases further in patients with poorly controlled HIV, who are subsequently more immunosuppressed. Interestingly, the risk of BCC has been shown to be increased only in the subset of HIV-infected patients who report men who have sex with men as their route of infection, possibly reflecting increased UV exposure in this subgroup [42,43].

In addition to the well-established risk associated with solid organ transplantation and HIV infection, newer targeted immunomodulatory therapies may also affect antitumour immunosurveillance in the skin. Janus kinase (JAK) inhibitors, which are increasingly used in dermatology for atopic dermatitis, psoriasis, alopecia areata and vitiligo, interfere with JAK-STAT signalling downstream of several cytokines essential for antitumour immunity, including interferons, IL-2 and IL-15, thereby potentially impairing the function of cytotoxic CD8+ T cells and NK cells [44]. In the ORAL Surveillance trial, which enrolled rheumatoid arthritis patients aged ≥50 years with at least one additional cardiovascular risk factor, tofacitinib was associated with a higher incidence of NMSC (hazard ratio [HR] 2.02), particularly cutaneous squamous cell carcinoma (HR 2.32), compared with TNF inhibitors [45,46]. Although meta-analyses of randomized controlled trials have not demonstrated an increased risk of NMSC compared with placebo [47,48], the follow-up periods were relatively short. Therefore, the use of systemic JAK inhibitors in elderly patients with pre-existing immunosenescence and cumulative sun-induced skin damage warrants caution, together with regular skin cancer screening.

Apart from the connection between NMSCs and the immune system through immunodeficiencies and immunosuppression, the correlation between the two is also evident through immunosenescence, a deterioration of the immune system caused by the aging process. Inflammation is a beneficial, acute, transient immune response to harmful conditions, but with aging the capacity to cope with antigenic, chemical, physical and nutritional stimuli declines. To protect tissue homeostasis, a persistent low-grade systemic inflammation, termed “inflammaging”, occurs, which stimulates a counteracting compensatory immunosuppression and leads to tissue dysfunction and degeneration [49,50]. It should be noted, however, that many immune-gerontologists caution against a purely negative reading of these changes: viewed from an evolutionary perspective, immunosenescence and inflammaging may be better understood as adaptive or remodelling processes rather than solely detrimental ones, and are increasingly regarded as closely linked and mutually reinforcing: the decline of adaptive immunity is accompanied by a compensatory, chronic activation of innate immunity that clears accumulating cellular debris. Cumulative data suggest that, without this immunosenescence–inflammaging interplay, human longevity would be considerably shortened [51]. In inflammaging, serum levels of inflammatory cytokines such as IL-6, IL-8, and TNF-α are elevated [52,53], resulting from activation of signalling networks critical to inflammation, such as those regulated by the Nuclear Factor (NF)-κB transcription factor, together with a variety of inflammatory stimuli that trigger and sustain inflammaging [54,55,56]. These cytokines drive innate immunity and are secreted by innate immune cells and non-immune cells that become dysfunctional with age (e.g., dendritic cells, neutrophils, macrophages, and fibroblasts) [57,58].

In the last two decades, substantial evidence has accumulated on the role of the inflammatory process and immunosenescence in promoting cancer. Not only can cancer arise at sites of chronic inflammation, but a pro-inflammatory microenvironment, supported by inflammatory cells and mediators, is also an essential component of cancer and one of its hallmarks [59,60,61,62,63]. Tumours also show an accumulation of senescent non-immune cells that can suppress tumour growth but can also enhance tumorigenesis through the proinflammatory senescence-associated secretory phenotype (SASP; detailed in Section 5).

At present, there is an ongoing debate as to whether immunosenescence is a cause or a consequence of inflammaging. Immunosenescence may be a consequence of inflammaging, since inflammatory diseases unrelated to aging show an extensive immunosenescent state of immune cells, e.g., many infections or autoimmune diseases [64,65]. Immunosenescence is also clearly increased in several age-related diseases, e.g., cardiovascular and neurodegenerative diseases [66,67]. Rheumatoid arthritis induces premature T cell senescence with deficient CD28 receptor expression and enhanced proinflammatory SASP properties [68,69], and neuropsychiatric disorders such as major depression and bipolar disorder are similarly associated with chronic inflammation and immunosenescence [70,71].

Psoriasis and hidradenitis suppurativa are common inflammatory and immune-mediated skin diseases characterized by increased levels of pro-inflammatory cytokines and chemokines such as TNF-α, IL-17 and IL-23. Several observational studies suggest that patients with these diseases are at increased risk of developing cancer [72,73,74,75], particularly keratinocyte skin cancer and lymphomas. Psoriasis patients have a higher skin cancer incidence than the general population, with a standardized incidence ratio of 3.37 (95% CI 1.84–5.66) [76], which could reflect inflammatory mediators that induce pro-cancerous mutations, modulate adaptive responses, promote resistance to apoptosis, and cause microenvironmental changes, but also different treatment options such as phototherapy and immunosuppressants [77,78].

Despite the many variables involved in assessing the influence of psoriatic therapy on skin cancer risk (predisposing factors, duration and timing of therapy, cumulative dose, interaction with other carcinogens, latency), sufficient evidence confirms a relation between skin cancer and specific psoriasis treatments [79]. Oral psoralen and ultraviolet A is associated with an increased, dose-dependent risk of skin cancer, and systemic non-biologic therapies such as methotrexate or cyclosporin are also associated with increased skin cancer risk [80], acting primarily as immunosuppressants. TNF-α inhibitors (infliximab, etanercept, adalimumab) are the oldest class of biologic drugs approved for psoriasis and, for adalimumab, hidradenitis suppurativa; since these drugs target molecules relevant to cancer immunosurveillance, concerns have been raised about a possible increased cancer risk [81,82,83,84,85]. A recent meta-analysis reported an increased risk of NMSC with TNF inhibitors in psoriasis patients, though the authors acknowledged significant limitations and a lack of real-world evidence [86], while another study found no increased risk of NMSC or melanoma with TNF-α inhibitors, ustekinumab, apremilast or tofacitinib, again with certain limitations [87].

Data also indicate a higher risk of NMSC in patients with hidradenitis suppurativa compared with the general population, alongside a greater overall risk of oral cavity, CNS, colorectal and prostate cancer [88]. Patients with bullous pemphigoid similarly show a higher risk of developing cutaneous SCC, most often after the first year of follow-up [89].

In summary, it remains difficult to determine whether the increased cancer risk in patients with psoriasis or hidradenitis suppurativa results from the disease itself and its inflammatory immune factors, from the effects of specific therapies, from a combination of both, or from additional contributing factors.

## 5. Cellular Senescence and Inflammaging in the Skin Microenvironment

Cellular senescence is a largely irreversible arrest of the cell cycle that follows intrinsic and extrinsic stresses such as telomere shortening, unrepaired DNA damage, mitochondrial dysfunction, oxidative stress, and oncogene activation [90,91]. These stresses activate a persistent DNA-damage response (DDR) in a way that the sensor kinase ATM stabilizes p53, which induces the cyclin-dependent kinase inhibitor p21 (CDKN1A) and halts the cell cycle, while sustained DDR signalling and reactive oxygen species (ROS) drive expression of p16INK4a (CDKN2A), acting through the RB pathway to maintain the arrest [92]. This state is self-reinforcing since the senescence-associated mitochondrial dysfunction generates further reactive oxygen species that sustain DNA damage and stabilize the senescent phenotype [93]. Although senescence originally evolved as a tumour-suppressive mechanism, halting the proliferation of cells at risk of malignant transformation, the progressive accumulation of senescent cells in aged tissue can instead promote, rather than restrain, carcinogenesis [91]. These opposing effects are linked by the SASP: senescent cells secrete pro-inflammatory cytokines (IL-1α, IL-1β, IL-6, IL-8), chemokines, growth factors, and matrix metalloproteinases (MMPs) that act on neighbouring cells and remodel the surrounding tissue [94]. Sustained, low-grade release of these mediators is a principal cellular driver of “inflammaging”, the chronic, sterile, systemic inflammation that characterizes the aging organism [95].

**Figure 2:**
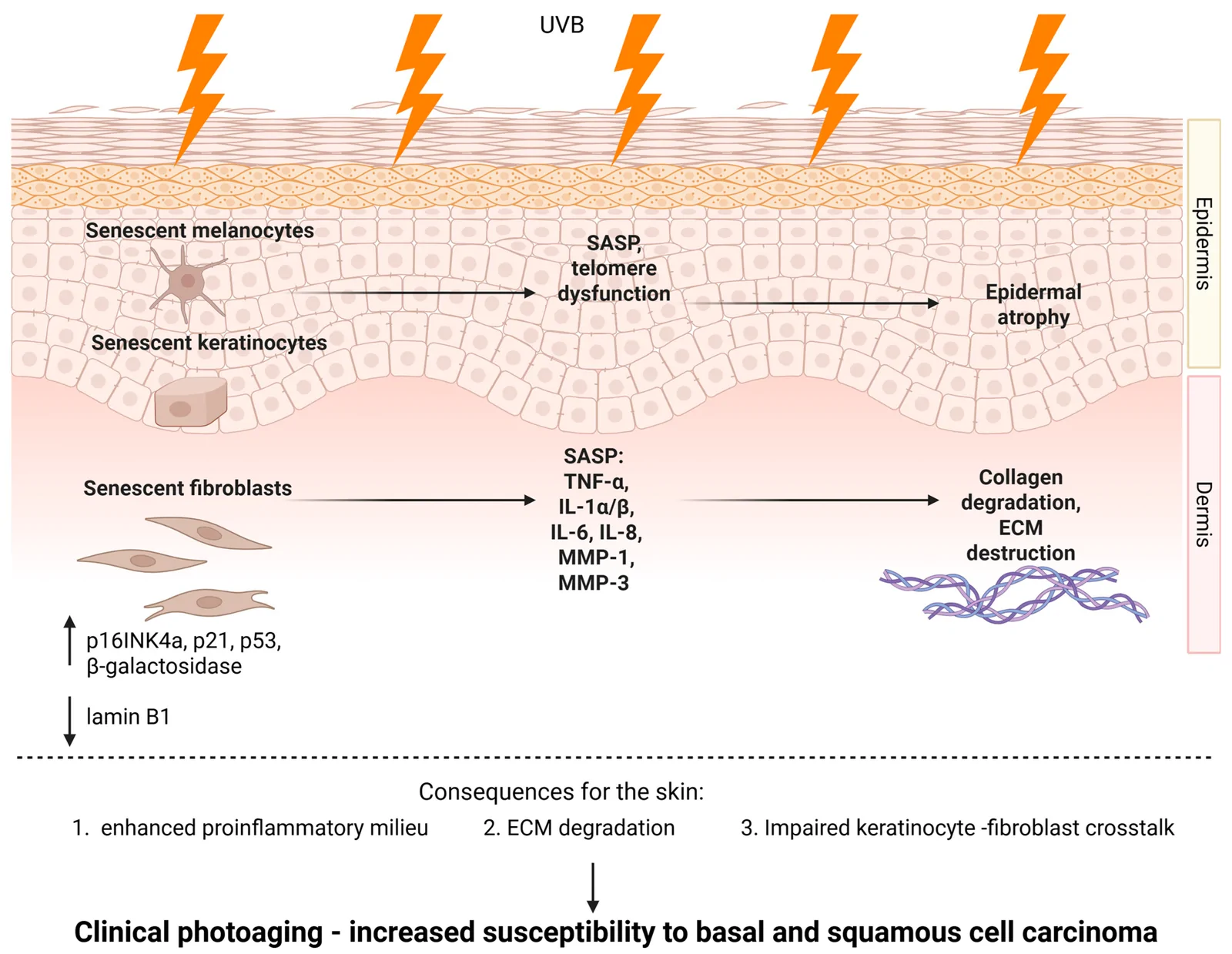
Cellular senescence and inflammaging in the skin microenvironment. Senescent keratinocytes, melanocytes, and dermal fibroblasts accumulate in aged and photoexposed skin, expressing p16INK4a, p21, p53 and SA-β-galactosidase while losing lamin B1. Their senescence-associated secretory phenotype (SASP) (IL-6, IL-8, IL-1α/β, TNF-α and MMP-1/3) drives collagen fragmentation and a self-amplifying loop of extracellular-matrix breakdown. mTOR (via IL-1α and MAPKAPK2), NF-κB, and UV-driven suppression of TGF-β/SMAD signalling govern this output, sustaining the low-grade inflammation termed inflammaging and predisposing to keratinocyte carcinoma. Abbreviations: SASP, senescence-associated secretory phenotype; IL, interleukin; TNF-α, tumour necrosis factor-α; MMP, matrix metalloproteinase; mTOR, mechanistic target of rapamycin; MAPKAPK2, MAPK-activated protein kinase 2; NF-κB, nuclear factor-κB; UV, ultraviolet; TGF-β, transforming growth factor-β; SA-β-galactosidase, senescence-associated β-galactosidase.

In the skin, which is directly exposed to external stressors, senescent cells accumulate in every major compartment. In the dermis, fibroblasts are considered the central hub of the senescence process [96]. Senescent dermal fibroblasts up-regulate the cyclin-dependent kinase inhibitors p16INK4a and p21, stabilize p53, and express senescence-associated β-galactosidase, while losing lamin B1 [90,96]. Functionally, their SASP is dominated by MMP-1 and MMP-3, which fragment types I and III collagen; the resulting collagen breakdown products further amplify oxidative stress and MMP expression, driving extracellular matrix (ECM) degradation [97]. Clinically, this manifests as the dermal thinning, loss of elasticity, and wrinkling that define photoaged and chronologically aged skin. The same ECM disruption that produces these visible changes also degrades the structural integrity that normally constrains epithelial cells, and senescence-driven remodelling of the aged dermal microenvironment impairs keratinocyte–fibroblast crosstalk and enhances susceptibility to basal and squamous cell carcinoma [96] (Figure 2).

**Figure 3:**
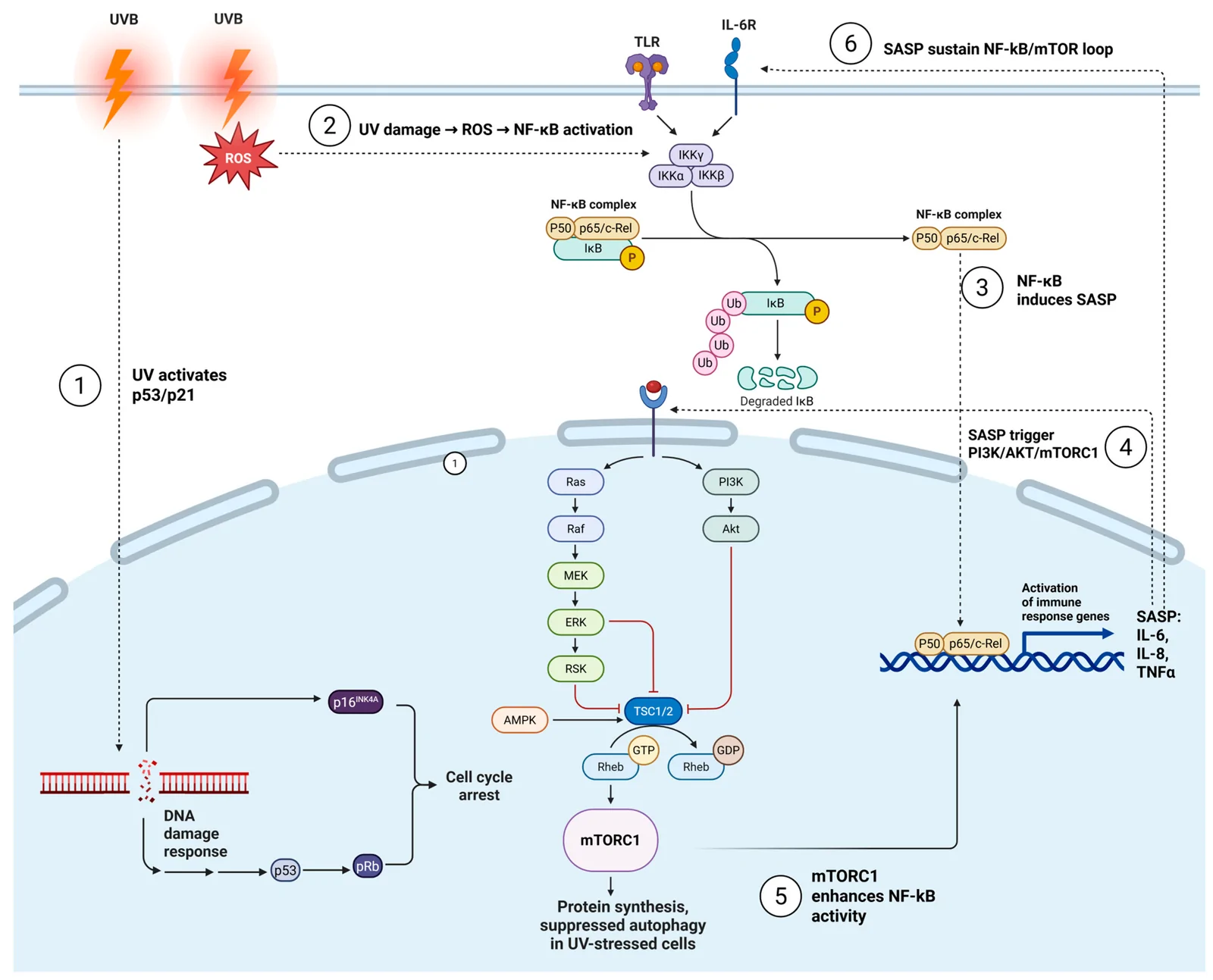
Molecular signalling loop linking DNA damage, NF-κB and mTORC1 in the senescence-associated secretory phenotype. UV radiation activates the p53/p21 axis of the DNA-damage response, halting the cell cycle. In parallel, UV-generated reactive oxygen species activate the IKK complex downstream of Toll-like receptors and the IL-6 receptor, triggering IκB degradation and nuclear translocation of the NF-κB complex (p50/p65/c-Rel), which induces transcription of SASP mediators (IL-6, IL-8, TNF-α). These secreted factors act in an autocrine/paracrine manner on Ras–Raf–MEK–ERK and PI3K–Akt signalling, converging on TSC1/2–Rheb to activate mTORC1; mTORC1 in turn enhances NF-κB transcriptional activity, and the resulting SASP output sustains continued TLR- and IL-6R-driven signalling, closing a self-amplifying NF-κB/mTOR loop that perpetuates senescent secretory output independently of the initiating stimulus. Abbreviations: DNA, deoxyribonucleic acid; NF-κB, nuclear factor-κB; mTORC1, mechanistic target of rapamycin complex 1; SASP, senescence-associated secretory phenotype; UV, ultraviolet; IKK, IκB kinase; TLR, Toll-like receptor; IL, interleukin; IL-6R, interleukin-6 receptor; TNF-α, tumour necrosis factor-α; MEK, MAPK/ERK kinase; ERK, extracellular signal-regulated kinase; PI3K, phosphoinositide 3-kinase; TSC, tuberous sclerosis complex.

Several signalling pathways govern this fibroblast SASP. The mechanistic target of rapamycin (mTOR) promotes SASP output both by driving translation of the membrane-bound cytokine IL-1α (an apical regulator of a broad NF-κB-dependent secretory programme) and by controlling MAPKAPK2 translation; accordingly, mTOR inhibition suppresses the SASP [98,99]. In parallel, UV radiation activates AP-1 and p53, which down-regulate TGF-β/SMAD signalling; because TGF-β normally sustains type I procollagen synthesis, its suppression simultaneously reduces collagen production and increases MMP-mediated collagen degradation, mechanistically coupling photoaging to the loss of dermal integrity that precedes cutaneous carcinogenesis [100,101] (Figure 3).

Senescence is not confined to fibroblasts. In the epidermis, keratinocytes exposed to UVB display the full senescence signature and secrete a SASP rich in TNF-α, IL-1α, IL-1β, and IL-6 [90]. Melanocytes are among the epidermal cell types that reliably express p16INK4a during intrinsic aging in sun-protected skin, and senescent melanocytes propagate senescence to neighbouring cells: through paracrine signalling they induce telomere dysfunction in surrounding keratinocytes and limit their proliferation, contributing to epidermal atrophy [102].

Xeroderma pigmentosum (XP) is an archetypal inherited condition that provides a particularly informative human model of premature cutaneous photoaging and accelerated photocarcinogenesis [103]. In most forms of this rare autosomal recessive disorder, defects in nucleotide excision repair (NER) compromise the removal of UV-induced cyclobutane pyrimidine dimers and 6–4 photoproducts. The XP variant differs mechanistically, arising from pathogenic variants in POLH that impair DNA polymerase η-mediated translesion synthesis despite preserved NER [104]. These abnormalities increase the persistence or mutagenic bypass of UV-induced DNA lesions, linking defective genome maintenance to early cutaneous damage and malignant transformation. Clinically, lentigines and poikiloderma, including pigmentary changes, telangiectasia and skin atrophy, can develop during childhood on sun-exposed sites [103,104]. The relative sparing of protected skin highlights the interaction between inherited susceptibility and environmental UV exposure [103]. In a long-term National Institutes of Health cohort of 106 patients, NMSC risk was approximately 10,000-fold higher in patients younger than 20 years than in the age-matched general population, while the median age at first NMSC diagnosis in the cohort was nine years [105]. Beyond epidermal mutagenesis, Fréchet et al. demonstrated increased oxidative stress and MMP-1 expression in XP-C dermal fibroblasts, with MMP-1 overexpression also detected in patient dermis [106]. These findings suggest that altered stromal function and matrix remodelling may contribute to both premature photoaging and a microenvironment that favours epithelial carcinogenesis [106]. XP therefore highlights defective genome maintenance as a link between premature cutaneous aging and photocarcinogenesis, complementing the mechanisms of senescence-associated stromal remodelling discussed above [103,106]. 

An additional mechanism contributing actively to the maintenance of senescence is mitochondrial dysfunction. Passos et al. showed that DDR signalling through p53 and p21 increases mitochondrial ROS production, and that these ROS cause further DNA damage, thereby stabilising the growth arrest [107]. Telomeric DNA is especially susceptible to such damage. Because telomere-bound shelterin proteins inhibit repair, DDR foci at telomeres can persist for months in ageing tissues and after stress-induced senescence [108]. The SASP also depends on mitochondria. In cells depleted of mitochondria, ROS production decreased and the expression of most pro-inflammatory SASP factors was markedly reduced, whereas the cell cycle arrest remained [93]. Vizioli et al. later described a retrograde pathway from mitochondria to the nucleus in which ROS-JNK signalling leads to the formation of cytoplasmic chromatin fragments. These fragments activate cGAS-STING and thus the SASP, and low-dose HDAC inhibitors reduced both effects by restoring mitochondrial function [109]. The metabolic state of the cell further shapes the secretome, as mitochondrial dysfunction with a low NAD+/NADH ratio induces a senescent phenotype that lacks the IL-1-dependent inflammatory arm [110]. In chronically photodamaged dermis this crosstalk between mitochondrial metabolism and chromatin seems to sustain MMP-1 and MMP-3 secretion by senescent fibroblasts and contributes to a stromal environment that favours keratinocyte transformation [111].

Together, these processes create a cutaneous microenvironment that is pro-inflammatory, matrix-degraded, and immunologically altered, favouring tumour initiation and progression. The persistent SASP sustains the low-grade inflammation that constitutes a recognized hallmark of cancer, while the accumulation of senescent stromal cells provides both the soluble mediators and the permissive matrix that support the outgrowth of transformed keratinocytes. Cellular senescence therefore provides a mechanistic link between visible photoaging and the increased incidence of non-melanoma skin cancer in older adults. The same SASP mediators that remodel the local stroma also act systemically on the immune compartment, contributing to the age-related decline in immune competence (immune senescence) considered in Section 8.

## 6. The Effect of UV-Light-Induced Skin Damage on Cutaneous Immune Function

UV radiation leaves a characteristic mutational footprint in keratinocyte carcinomas. Aggressive cSCC carries one of the highest mutation rates reported in human cancer, dominated by C > T transitions at dipyrimidine sites [112,113]. Such tumours should be highly immunogenic, and the response rate of approximately 50% to cemiplimab in advanced cSCC supports this [114]. Yet many NMSC escape T-cell recognition. As mentioned in previous sections, BCC often shows reduced HLA class I and β2-microglobulin expression [115], so a large neoantigen repertoire is of little use when it cannot be presented. Loss of HLA class I, in turn, should expose tumour cells to NK cells, which kill targets lacking self-MHC (‘missing self’) [116]. The clinical relevance of this mechanism is supported by the observation that low circulating NK cell counts, together with high regulatory T-cell numbers, were associated with a more than five-fold higher risk of cSCC in kidney transplant recipients [117]. In older individuals this second line is also weakened: NK cells from elderly donors release less perforin per cell [118]. BCC cells can additionally shed soluble CD200, which suppresses IFN-γ production by NK cells and drives their apoptosis [119]. Together, these observations suggest that in the ageing skin a high UV-induced mutational load meets a simultaneous failure of T-cell and NK-cell surveillance.

Beyond its well-established mutagenic action, ultraviolet radiation (UVR) is a potent immunosuppressant, and this immunomodulatory property is now regarded as an independent and essential co-factor in cutaneous carcinogenesis rather than a mere epiphenomenon of DNA damage [120]. In classic experiments, highly antigenic UV-induced tumours grew unimpeded in a UV-conditioned host but were rejected in a naïve immunocompetent one, first revealing that UVR can disable tumour immunosurveillance [121]. UV-induced immunosuppression occurs at suberythemogenic, physiological doses, so the immune effects of sun exposure occur well below the threshold of visible sunburn [122].

To exert its immunological effects, UVR must first be absorbed by an epidermal chromophore and converted into a biological signal. Two principal photoreceptors have been identified: nuclear DNA and trans-urocanic acid (UCA). Direct absorption of UVB by DNA generates cyclobutane pyrimidine dimers (CPDs) and 6–4 photoproducts, which, besides being mutagenic, also trigger immunosuppression; enzymatic photorepair of CPDs in irradiated antigen-presenting cells restores their capacity to present antigen and abrogates the immunosuppressive effect, establishing CPDs as a causal upstream event [123]. In parallel, UVR isomerizes trans-UCA, abundant in the stratum corneum, to its cis-isomer; cis-UCA is a well-characterized soluble mediator of UV immunosuppression that signals in part through the serotonin (5-HT2A) receptor and inhibits the ability of Langerhans cells to present tumour antigens, thereby directly linking a UV photoproduct to impaired anti-tumour immunity [124,125].

These molecular events converge on the cellular effectors of cutaneous immunity. Epidermal Langerhans cells and dermal dendritic cells (the sentinel antigen-presenting cells of the skin) are central targets. Following UV exposure they sustain DNA damage that impairs their antigen-presenting function, reduces their density and migratory capacity, and reprograms them from immunogenic into tolerogenic antigen-presenting cells [120,126]. Rather than priming effector responses, these UV-conditioned dendritic cells preferentially drive the generation of regulatory T cells (Tregs) [126,127]. This shift is amplified by keratinocytes and skin-infiltrating macrophages, which respond to UVR by secreting immunosuppressive cytokines (most notably interleukin-10) alongside the pro-inflammatory mediators TNF-α and IL-1β, biasing the cutaneous response from a protective T helper 1 (Th1) toward a permissive Th2 profile [120].

The end result is an antigen-specific, transferable state of immune tolerance mediated by regulatory T cells [122]. Whereas this mechanism may have evolved to limit autoimmune responses against UV-modified self-antigens, in the context of photocarcinogenesis it is harmful: the same Tregs that suppress contact hypersensitivity also suppress the anti-tumour response, permitting nascent, antigenic keratinocyte clones to escape immune elimination [121,125]. This links sun exposure to the clinical observation that cutaneous SCC, the most antigenic and immune-dependent of the keratinocyte cancers, arises preferentially at chronically sun-exposed sites and is markedly over-represented in states of impaired immunosurveillance.

**Figure 4:**
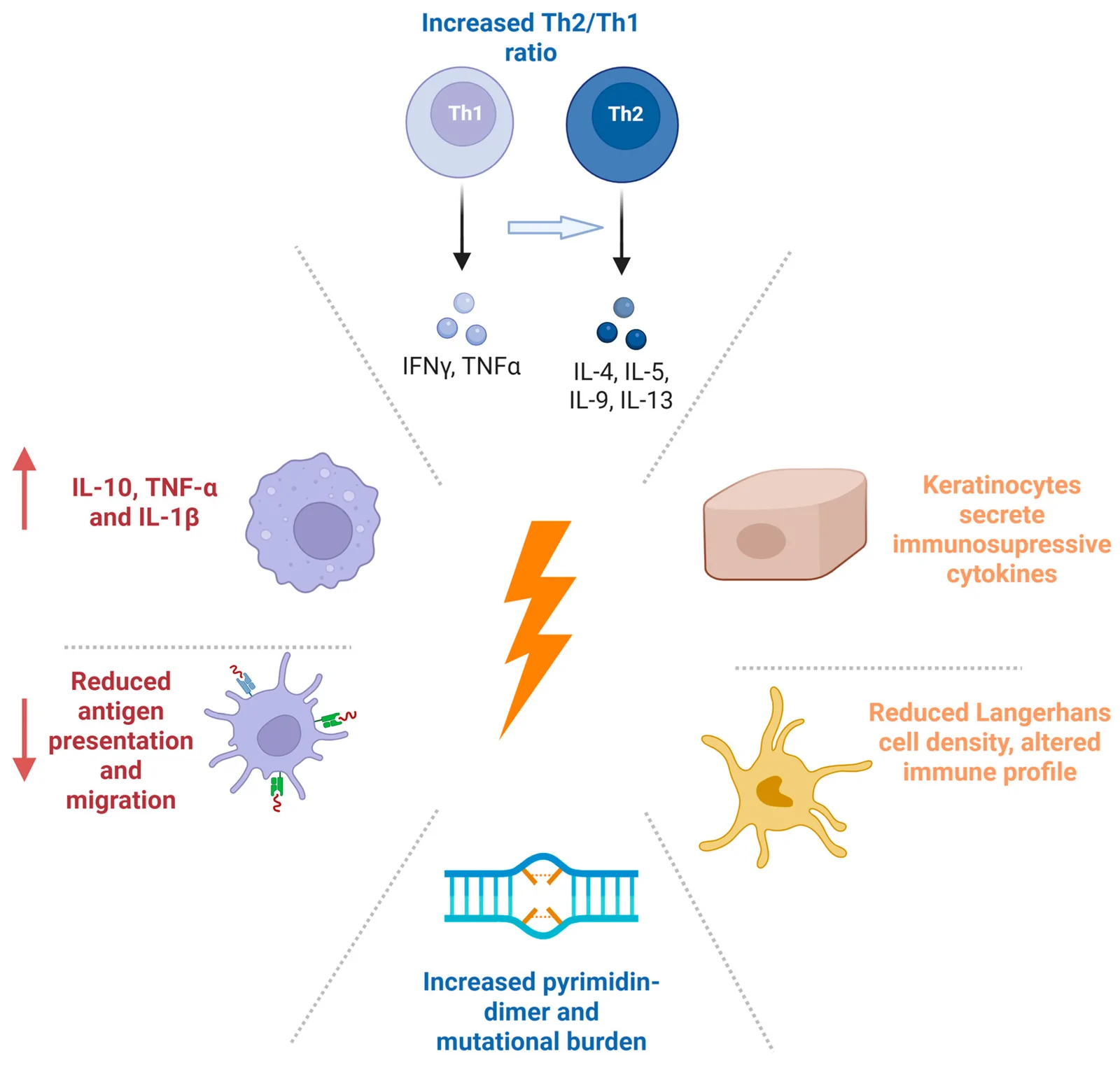
UV-induced suppression of cutaneous immune function. UV exposure increases pyrimidine-dimer formation and cutaneous mutational burden, reduces Langerhans cell density, and impairs Langerhans cell antigen presentation and migration. Keratinocytes and macrophages respond by secreting the immunosuppressive cytokine IL-10 alongside the pro-inflammatory mediators TNF-α and IL-1β, while the cutaneous cytokine balance shifts from a protective Th1 profile (IFN-γ, TNF-α) toward a permissive Th2 profile (IL-4, IL-5, IL-9, IL-13). Together, these changes permit escape of antigenic keratinocyte clones and impair surveillance of cutaneous SCC, additive to the baseline immunosenescent phenotype of aged skin. Abbreviations: UV, ultraviolet; IL, interleukin; TNF-α, tumour necrosis factor-α; IFN-γ, interferon-γ; Th, T helper; SCC, squamous cell carcinoma.

UV-induced immunosuppression does not act in isolation; it is superimposed on the age-related immune decline described above. The aged cutaneous immune system already exhibits reduced Langerhans cell numbers, blunted dendritic-cell function, a contracted naïve T-cell repertoire, and an expanded regulatory compartment: the immunosenescent phenotype [51,128]. UVR pushes the immune system in the same direction, so that in chronically sun-exposed elderly skin the acute immunosuppressive burden of each UV insult is added to a baseline of diminished immune competence. This convergence of extrinsic photoimmunosuppression and intrinsic immunosenescence plausibly explains why the incidence of NMSC rises so steeply with the combination of advancing age and cumulative sun exposure, and is consistent with the integrated model developed in Section 8 (Figure 4).

## 7. The Role of Skin Dysbiosis in Shaping Immune Responses Against Microbes and Tumors

The skin hosts a diverse and largely commensal microbial community that actively contributes to cutaneous immune homeostasis. Beyond providing colonization resistance against pathogens, resident microbes continuously educate and calibrate the skin immune system, and increasing evidence implicates disruption of this equilibrium (dysbiosis) as a further contributor to NMSC pathogenesis, alongside UV mutagenesis and immune decline [129].

The immunomodulatory capacity of the commensal microbiota is best exemplified by Staphylococcus epidermidis, the dominant Gram-positive colonizer of healthy skin. *S. epidermidis* reinforces barrier integrity, in part by secreting a sphingomyelinase that supplies ceramides, the principal barrier lipids whose depletion drives skin dehydration and aging, and simultaneously tunes local immunity [130]. Specific strains of *S. epidermidis* induce commensal-specific CD8+ T cells across an intact, uninflamed epidermis, a form of adaptive immunity generated in the absence of infection. A distinct *S. epidermidis* strain producing the nucleobase analogue 6-N-hydroxyaminopurine suppresses UV-induced cutaneous tumour formation by selectively inhibiting DNA synthesis in transformed cells [131]. The healthy microbiome therefore contributes an anti-neoplastic function, coupling barrier maintenance to active tumour immunosurveillance.

Dysbiosis, by contrast, favours keratinocyte carcinogenesis. Studies profiling the microbial composition of healthy skin, actinic keratosis, and cutaneous SCC have documented a characteristic compositional shift, most consistently a depletion of the potentially protective Cutibacterium acnes accompanied by enrichment of the pathobiont *Staphylococcus aureus* [132,133,134]. *S. aureus* actively manipulates host immunity and secretes toxins and virulence factors that sustain chronic inflammation, and its expansion is associated with elevated cutaneous SCC risk [132]. The resulting inflammatory, immunosuppressive niche both fuels tumour-promoting inflammation and blunts the commensal-dependent CD8+ T-cell surveillance that normally restrains transformed keratinocytes, so that microbial imbalance and local immune failure reinforce one another [132].

The functional consequences of skin dysbiosis extend to therapeutic response: modulation of the microbiome alters the efficacy of immune checkpoint inhibition in cutaneous SCC models, demonstrating that the cutaneous microbial community can influence not only tumour initiation but also the outcome of immunotherapy [135]. This parallels the well-established role of the gut microbiome in shaping checkpoint-inhibitor responses in melanoma and underscores the microbiome as a potentially modifiable determinant of NMSC behaviour.

Two features make skin dysbiosis particularly relevant here. First, the aged skin surface undergoes measurable shifts in microbial composition and diversity, driven by changes in sebaceous output, pH, barrier function, and local immunity: the same barrier-degrading and immunosuppressive processes generated by cellular senescence and photoaging described in the preceding sections [136,137,138]. Reported age-related changes include a rise in overall community diversity accompanied by a relative depletion of *Cutibacterium* and expansion of *Corynebacterium*, *Streptococcus* and *Staphylococcus species*, alongside the more alkaline, less hydrated surface of aged skin that favours opportunistic colonization. Second, the anti-tumour arm of commensal immunity is itself immune-cell dependent: the CD8+ T-cell responses elicited by beneficial commensals rely on the same effector T-cell compartment that is eroded by immunosenescence. Age-related dysbiosis and immunosenescence are therefore not independent risk factors but mutually reinforcing, together creating a microenvironment that is permissive to non-melanoma skin cancer (Figure 1).

## 8. The Possible Role of Immune Senescence in the Aetiopathogenesis of Non-Melanoma Skin Cancer

The preceding sections establish three parallel, age-associated processes in the skin: the accumulation of senescent, SASP-secreting stromal cells (Section 5), the immunosuppressive reprogramming imposed by cumulative UV exposure (Section 6), and the dysbiotic shift in the cutaneous microbiome (Section 7). A growing body of work suggests that immune senescence, the systemic age-related remodelling of innate and adaptive immunity, provides a permissive background in which these local processes can progress to non-melanoma skin cancer. Within this framework, immunosenescence may act less as an isolated risk factor than as a common pathway through which accumulated mutational and inflammatory damage progresses to malignancy [139,140].

The main mechanism is the age-related erosion of the T-cell compartment that mediates cutaneous tumour immunosurveillance. With age, thymic involution reduces naïve T-cell output, contracting and skewing the T-cell receptor repertoire and impairing responses to newly encountered antigens, such as the neoantigens of a nascent tumour [141]. At the same time, chronic antigenic stimulation leads to the accumulation of terminally differentiated, oligoclonal memory cells that progressively lose the co-stimulatory molecules CD27 and CD28 while up-regulating CD57 and KLRG1; these CD28null senescent T cells have shortened telomeres, limited proliferative capacity, and increased resistance to apoptosis [142]. Because full activation of naïve tumour-specific CD8+ T cells requires CD28 co-stimulation, its loss directly compromises the priming of anti-tumour responses [143].

Clinically, impaired T-cell immunity is strongly associated with NMSC incidence. The 15- to 250-fold excess of cutaneous SCC in solid-organ transplant recipients, who are pharmacologically deprived of effective T-cell surveillance, is the strongest clinical evidence that cell-mediated immunity controls [33]. Functional studies confirm the mechanism: CD8+ cytotoxic T cells, rather than CD4+ or natural killer (NK) cells, are essential for cutaneous SCC regression, and their intratumoural infiltration depends on IFN-γ-induced chemokine expression [144]. Higher densities of intratumoural CD8+ tumour-infiltrating lymphocytes correlate with smaller tumour size, lower stage, absence of metastasis, and improved disease-free survival in cutaneous SCC [145]. Cutaneous SCC is therefore an immunologically “hot”, immune-dependent tumour, and its occurrence reflects failure of the same effector compartment that immunosenescence erodes.

The most direct evidence linking immunosenescence to NMSC comes from a study of elderly kidney transplant recipients, in which the exhaustion and defective differentiation of CD8+ central memory responder T cells preceded and predicted the development of NMSC and its precursor lesions [146]. This is important because it shows that a senescent, exhausted T-cell phenotype is not only a correlate of aging but a measurable antecedent of keratinocyte carcinogenesis, linking chronological age, pharmacological immunosuppression, and NMSC risk.

Immunosenescence also reshapes the innate arm and the tumour microenvironment in tumour-permissive ways. Antigen presentation by dendritic cells declines, NK-cell cytotoxicity wanes, and the numbers and suppressive activity of regulatory T cells and myeloid-derived suppressor cells increase, while the SASP sustains a chronically inflamed, immunosuppressive milieu [139,140]. This aging tumour microenvironment differs fundamentally from that of younger skin: it combines diminished effector capacity with increased suppressive and pro-inflammatory signalling, as seen in the senescent skin described throughout this review.

**Figure 5:**
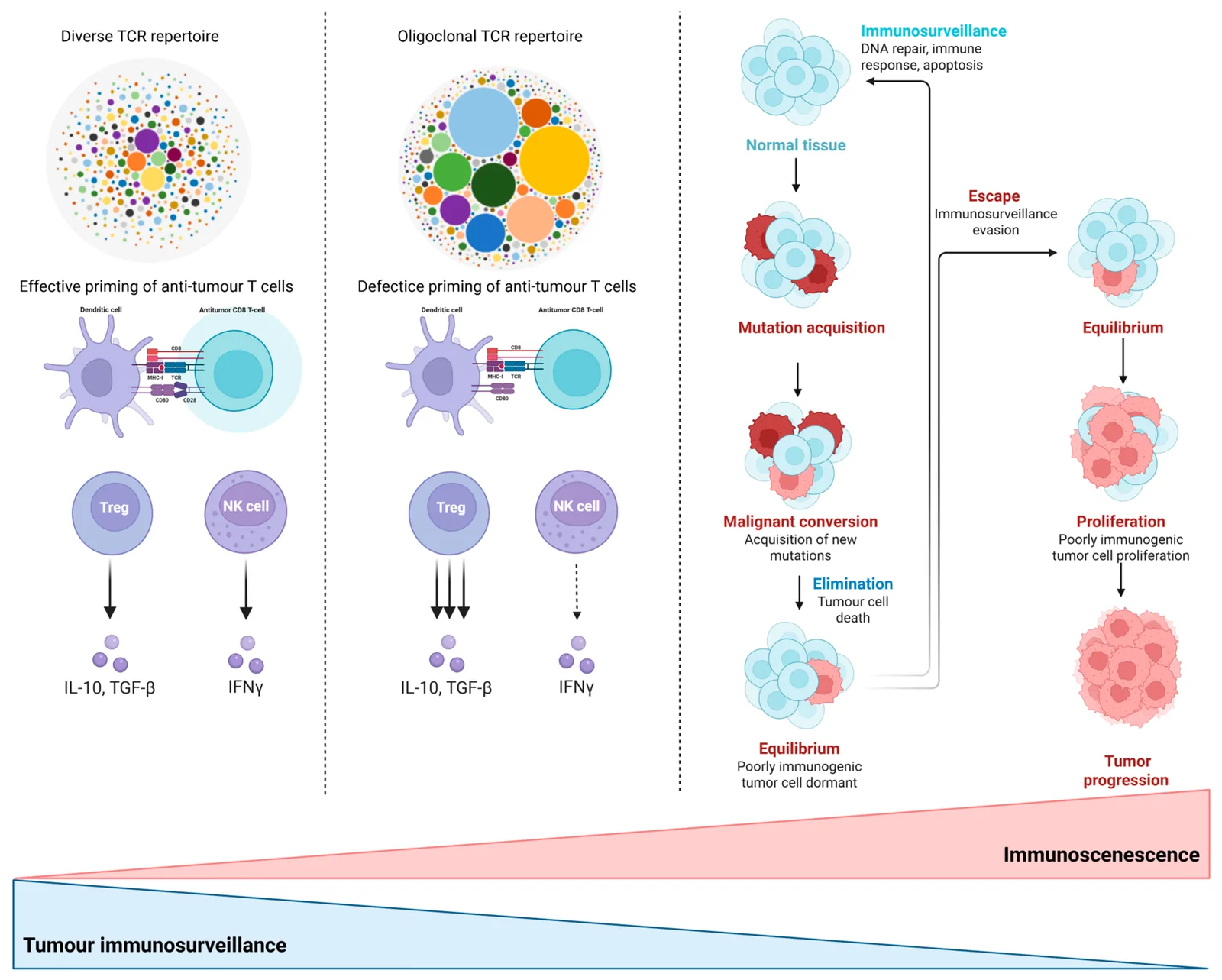
T-cell immunosenescence and loss of NMSC immunosurveillance. Thymic involution contracts the naive T-cell repertoire, while chronic antigenic stimulation drives accumulation of terminally differentiated CD28null/CD57+/KLRG1+ T cells with short telomeres and apoptosis resistance. Loss of CD28 co-stimulation impairs priming of tumour-specific CD8+ T cells; NK cytotoxicity declines and regulatory/myeloid-derived suppressor populations expand. A diverse T-cell receptor repertoire enables effective dendritic-cell priming of antitumour CD8+ T cells via CD80–CD28 co-stimulation, whereas its oligoclonal contraction yields defective priming and a skew toward Treg- and NK-derived IL-10/TGF-β output over IFN-γ. This decline is depicted across the classical cancer immunoediting cycle (elimination, equilibrium, and escape) in which poorly immunogenic tumour cells that evade elimination persist in equilibrium before proliferating and progressing as immunosenescence advances and tumour immunosurveillance wanes. Clinical evidence—the 65–250-fold cSCC excess in transplant recipients, the prognostic value of CD8+ TILs, and a cohort in which exhausted CD8+ central-memory T cells preceded NMSC in elderly kidney transplant recipients—supports immunosenescence as the linchpin converting accumulated cutaneous damage into clinically overt malignancy. Abbreviations: NMSC, non-melanoma skin cancer; NK, natural killer; Treg, regulatory T cell; TGF-β, transforming growth factor-β; IFN-γ, interferon-γ; IL, interleukin; cSCC, cutaneous squamous cell carcinoma; TIL, tumour-infiltrating lymphocyte.

Overall, these observations suggest the following model. Cumulative UV exposure generates both the initiating mutations and, through photoimmunosuppression, an early impairment of local tumour surveillance. Cellular senescence in the dermis and epidermis degrades the extracellular matrix and floods the microenvironment with pro-inflammatory, matrix-remodelling SASP factors. Age-related dysbiosis removes the protective, commensal-dependent CD8+ surveillance and enriches pro-inflammatory pathobionts. Underlying all of these, systemic immunosenescence, characterized by contraction of the naïve repertoire and accumulation of exhausted CD28null effector cells, may remove a final check on malignant outgrowth. No single process may be sufficient on its own, but together, in the same aged host, they could help explain why NMSC incidence rises so disproportionately with age, beyond what UV mutational burden alone would predict. In this view, immunosenescence may be the common factor that allows accumulated cutaneous damage to progress to clinically apparent non-melanoma skin cancer (Figure 5).

## 9. Measures to Prevent Non-Melanoma Skin Cancer

### 9.1. Classical Preventive Strategies

The cornerstone of NMSC prevention remains the reduction in cumulative and intermittent ultraviolet exposure. Primary prevention rests on well-established behavioural measures (sun avoidance during peak-irradiance hours, protective clothing and hats, and regular application of broad-spectrum sunscreen) reinforced by public-health education and the avoidance of artificial tanning (Table 1). Secondary prevention centres on the early detection and treatment of precursor lesions, principally actinic keratoses and Bowen’s disease, through field-directed therapies (topical 5-fluorouracil, imiquimod, photodynamic therapy) and lesion-directed cryotherapy, together with structured surveillance of high-risk individuals: organ transplant recipients, patients with prior NMSC, and those with genetic predisposition. Among pharmacological chemopreventive agents, oral nicotinamide (vitamin B3) has the strongest evidence base: in the phase 3 ONTRAC randomized trial, 500 mg twice daily reduced new NMSC and actinic keratoses in high-risk but immunocompetent patients, an effect attributed to enhanced repair of UV-induced DNA damage, restoration of cellular ATP, and attenuation of UV-induced cutaneous immunosuppression [147,148]. Other agents with clinical evidence include oral retinoids, particularly in transplant recipients [148].

### 9.2. Rethinking Prevention in the Light of Immune Senescence and Inflammaging

The mechanistic framework developed in this review suggests that prevention strategies directed solely at UV exposure, while necessary, are incomplete for the elderly patient in whom immunosenescence, cellular senescence, and dysbiosis have already reshaped the cutaneous microenvironment. If age-related immune decline is a central and partly modifiable determinant of NMSC, then complementary strategies aimed at the aged immune system merit serious consideration (Table 1).

Several observations support this reorientation. First, the differential efficacy of nicotinamide is itself instructive: whereas nicotinamide reduces NMSC in immunocompetent individuals, the ONTRANS trial found no significant benefit in immunosuppressed organ transplant recipients [128]. This dissociation implies that in a host with severely compromised T-cell surveillance (a pharmacological model of the immunosenescent state) enhancing DNA repair and relieving photoimmunosuppression is insufficient, and reinforces the idea that the integrity of adaptive immunity is rate-limiting for chemoprevention.

Second, senotherapeutics offer a further, complementary approach. Because the accumulation of senescent, SASP-secreting fibroblasts and keratinocytes contributes both to the pro-inflammatory microenvironment and to the matrix remodelling that favours keratinocyte carcinogenesis, the selective elimination of senescent cells (senolytics, for example the combination of dasatinib and quercetin, the BCL-2/BCL-xL inhibitor navitoclax, or the flavonoid fisetin) or the suppression of their secretory phenotype (senomorphics, such as rapamycin and other mTOR inhibitors, metformin, or JAK inhibitors that dampen SASP signalling) has been proposed as a strategy not only for skin rejuvenation but potentially for the prevention of precancerous lesions such as actinic keratoses [149,150]. The clinical evidence remains early but is encouraging. For the oral senolytic combination dasatinib plus quercetin, an initial human study in patients with diabetic kidney disease demonstrated that a short, intermittent course reduced the senescent-cell burden (including p16INK4A- and p21CIP1-expressing cells and cells with senescence-associated β-galactosidase activity) in adipose tissue and skin, establishing proof of principle that senescent cells can be cleared in humans with an acceptable short-term safety profile [151]. Of greater dermatological relevance, a topical, and therefore low-systemic-exposure, approach may be feasible: in an exploratory placebo-controlled trial, daily application of a low-concentration topical rapamycin (a senomorphic mTOR inhibitor) over several months reduced epidermal p16INK4A expression and the histological hallmarks of photoaging, including solar (actinic) elastosis, a change directly relevant to the actinic-keratosis field [152]. Together with mechanistic data showing that rapamycin blocks IL-1α-driven SASP output and attenuates UVB-induced senescence in dermal fibroblasts, these findings identify topical senomorphics as a plausible, well-tolerated route for field prevention, while systemic senolytics remain, for now, experimental in this indication [149,150]. Targeting inflammaging at its cellular source could, in principle, interrupt the link between senescence, chronic inflammation, and malignant transformation described in Section 5.

Nevertheless, the potential senomorphic benefits of JAK inhibitors must be balanced against the risk that systemic treatment may suppress antineoplastic immunosurveillance. Ruxolitinib has been shown to reduce natural killer-cell numbers and impair cytokine-dependent activation and cytotoxicity, providing a plausible mechanism for weakened immune control of transformed cells [153]. A similar signal was reported for tofacitinib in the ORAL Surveillance trial, as discussed above [46]. A retrospective cohort study also linked ruxolitinib exposure to increased NMSC risk, particularly cSCC, in patients with polycythaemia vera or myelofibrosis [154]. Systemic JAK inhibitors should therefore not be presented as established NMSC-preventive senomorphics, and their use for other indications warrants drug- and patient-specific cancer risk assessment and appropriate dermatological surveillance [46,154].

Third, the recognition that the cutaneous microbiome actively shapes anti-tumour immunity, and that age-related dysbiosis erodes commensal-dependent CD8+ surveillance (Section 7), raises the prospect of microbiome-directed prevention: restoring protective commensals or their anti-neoplastic metabolites, or correcting the pathobiont enrichment associated with elevated cutaneous SCC risk. Because the cutaneous microbiome also modulates the response to immune checkpoint inhibition [135], such approaches may hold relevance both for prevention and for the optimization of established immunotherapy.

Several pilot studies in kidney transplant recipients support the concept of immune-stratified surveillance. CD4+ T-cell lymphopenia was among the first immune parameters associated with an increased risk of skin cancer in this population [155]. In a cohort of 116 kidney transplant recipients, the combination of high circulating regulatory T-cell and low NK cell counts was associated with a more than five-fold higher risk of cSCC [117]. In a subsequent prospective study of 117 high-risk recipients, a CD8+ immunosenescent phenotype, defined as more than 50% of CD8+ T cells expressing CD57, independently predicted new cSCC (adjusted HR 2.85), whereas the clinical risk scores lost their predictive value after their age component was removed [156]. Although these markers have not yet been validated in larger or non-transplant cohorts, they suggest that immunosenescence-related biomarkers could help identify patients who would benefit from intensified dermatological surveillance or modification of immunosuppression, such as conversion to mTOR inhibitors, which reduced the risk of secondary cSCC in kidney transplant recipients [157].

Finally, these considerations argue for a stratified, immune-aware approach to prevention in older adults. Rather than applying uniform, UV-centric measures, prevention could be tailored to an individual’s immune status, with intensified surveillance and, potentially, immune-directed intervention for those exhibiting markers of advanced immunosenescence (contraction of the naïve T-cell repertoire, expansion of CD28null effector cells, or a defective central-memory compartment). This aligns with the earlier proposal (Section 3) to reclassify recurrent NMSC as a chronic disease warranting long-term, coordinated management, and it reframes NMSC prevention in the elderly as, in part, a problem of managing the aging immune system.

**Table 1:** Classical and immune-senescence-informed measures for the prevention of non-melanoma skin cancer.

Preventive Measure/Agent	Mechanism of Action	References
**Classical preventive strategies**		
Photoprotection (sun avoidance, protective clothing/hats, broad-spectrum sunscreen; avoidance of artificial tanning)	Reduces cumulative and intermittent UV exposure, limiting direct DNA photodamage and UV-induced cutaneous immunosuppression	[158,159]
Field-directed treatment of precursor lesions (topical 5-fluorouracil, imiquimod, photodynamic therapy)	Eliminates actinic keratoses and Bowen’s disease over field-cancerized skin before progression to invasive carcinoma	[160]
Lesion-directed cryotherapy	Physical ablation of individual precursor lesions	[160]
Structured surveillance of high-risk groups (transplant recipients, prior NMSC, genetic predisposition)	Enables early detection and treatment, reducing tumour burden and morbidity	[161]
Oral nicotinamide (vitamin B3), 500 mg twice daily	Enhances repair of UV-induced DNA damage, restores cellular ATP, and attenuates UV-induced cutaneous immunosuppression; phase 3 ONTRAC trial showed reduced NMSC and actinic keratoses in immunocompetent patients (no benefit in transplant recipients, ONTRANS)	[128,147,148]
Oral retinoids	Modulate keratinocyte proliferation and differentiation; clinical evidence mainly in organ transplant recipients	[148]
**Immune-senescence- and inflammaging-informed strategies (proposed/emerging)**		
Senolytics: dasatinib + quercetin; navitoclax (BCL-2/BCL-xL inhibitor); fisetin	Selectively eliminate senescent cells, reducing SASP-driven pro-inflammatory, matrix-remodelling microenvironment; oral dasatinib + quercetin reduced p16INK4A-/p21CIP1-positive senescent cells in human skin and adipose tissue with acceptable short-term safety	[149,150,151]
Senomorphics: topical rapamycin (sirolimus); other mTOR inhibitors; metformin; JAK inhibitors (with caution, given potential impairment of antitumour immunosurveillance)	Suppress the SASP without cell killing; low-concentration topical rapamycin reduced epidermal p16INK4A and histological photoaging (solar/actinic elastosis) in a placebo-controlled trial; blocks IL-1α-driven SASP and attenuates UVB-induced fibroblast senescence, a well-tolerated, low-systemic-exposure route for field prevention	[149,150,152]
Microbiome-directed prevention (restoration of protective commensals/anti-neoplastic metabolites; correction of pathobiont enrichment)	Re-establishes commensal-dependent CD8+ antitumour surveillance and reduces pro-inflammatory pathobiont burden; the cutaneous microbiome also modulates response to immune checkpoint inhibition	[135]
Immune-stratified surveillance and intervention	Tailors prevention to individual immune status using markers of advanced immunosenescence (naïve T-cell contraction, CD28null effector expansion, defective central-memory compartment)	[117,155,156,157]

NMSC, non-melanoma skin cancer; SCC, squamous cell carcinoma; SASP, senescence-associated secretory phenotype; mTOR, mechanistic target of rapamycin; ATP, adenosine triphosphate; ONTRAC/ONTRANS, Oral Nicotinamide to Reduce Actinic Cancer trials. cSCC, cutaneous squamous cell carcinoma; NK, natural killer; Treg, regulatory T cell. Dashes indicate measures for which no single citation is assigned in the text.

## 10. Conclusions

Non-melanoma skin cancer is conventionally understood as the predictable consequence of a lifetime of ultraviolet exposure. The evidence reviewed here suggests that this account, while accurate, is incomplete. The steep, disproportionate rise in NMSC incidence with advancing age may be better explained not by cumulative mutation alone but by the combined effect of cellular senescence, photoimmunosuppression, cutaneous dysbiosis and, underlying all of these, systemic immunosenescence, which together weaken the tumour-surveillance mechanisms that would otherwise contain transformed keratinocytes. UV radiation and aging do not act separately in this process; they reinforce each other. Chronic sun exposure speeds up immunosenescence and the senescence of dermal cells, and aged skin in turn repairs UV damage less effectively. If immunosenescence is a central and partly modifiable factor in NMSC aetiopathogenesis, these cancers may be viewed as a disease of the aging immune system as much as of the aging skin. This perspective is consistent with the clinical epidemiology of NMSC in the elderly and suggests preventive and therapeutic options (senotherapeutics, microbiome modulation and immune-stratified surveillance) that go beyond UV-directed measures alone. Testing this will require prospective studies correlating validated markers of immunosenescence with NMSC risk, and interventional trials examining whether modulation of the aging immune system can reduce the burden of the most common malignancies in humans.

## Data Availability

No new data were created or analyzed in this study. Data sharing is not applicable to this article.
